# Global knowledge mapping and emerging research trends in non-coding RNAs related to animal and plant male sterility: A visual analysis of CiteSpace maps

**DOI:** 10.1097/MD.0000000000042612

**Published:** 2025-06-06

**Authors:** Mingzhao Zhang, Baojun Ju, Xiangyu Wang, Shuxi Zhou, Haobin Zhao, Zu long Wang, Xiao Li

**Affiliations:** a Department of Andrology, The First Affiliated Hospital, Henan University of Chinese Medicine, Zhengzhou, Henan, China; b Department of Clinical Medicine, The First Affiliated Hospital, Henan University of Chinese Medicine, Zhengzhou, Henan, China; c Department of Clinical Medicine, Henan University of Chinese Medicine, Zhengzhou, Henan, China.

**Keywords:** gene regulation, male sterility, model organism, ncRNA, RNA sequencing

## Abstract

Animal and plant male sterility is a complex and closely studied phenomenon that significantly impacts species survival and reproduction. Advances in biotechnology and molecular biology have deepened our understanding of gene expression regulation, particularly the role of noncoding RNAs (ncRNAs). This study aims to systematically review and analyze the published literature on ncRNAs in relation to both animal and plant sterility using bibliometric methods. A bibliometric analysis was conducted with CiteSpace 6.2.R6, Scimago Graphica, VOSviewer 1.6.18, and Microsoft Excel 2016 to identify research hotspots, key developments, and emerging trends. Data were retrieved from the Web of Science Core Collection on March 3, 2024, covering publications from 2005 to 2023. The analysis revealed a consistent increase in annual publications on ncRNA research in both plant and animal fields, with China and the United States leading in publication volume. Notable scholars include Professor Abu-Halima, a prominent figure in ncRNA research related to animal male sterility, and Professor Meyers, a key contributor to plant male sterility research. Journals such as PLoS ONE serve as major platforms for disseminating findings on animal male sterility, while The Plant Cell plays a similar role for plant male sterility. Analysis of cited literature and keyword trends highlighted significant themes, including gene regulation and the application of novel technologies. At present, new technologies, model organisms, and gene regulation remain major research hotspots. Meanwhile, disease diagnosis, disease treatment, and crop improvement are emerging as important directions for future research.

## Graphical abstract

**Figure:**
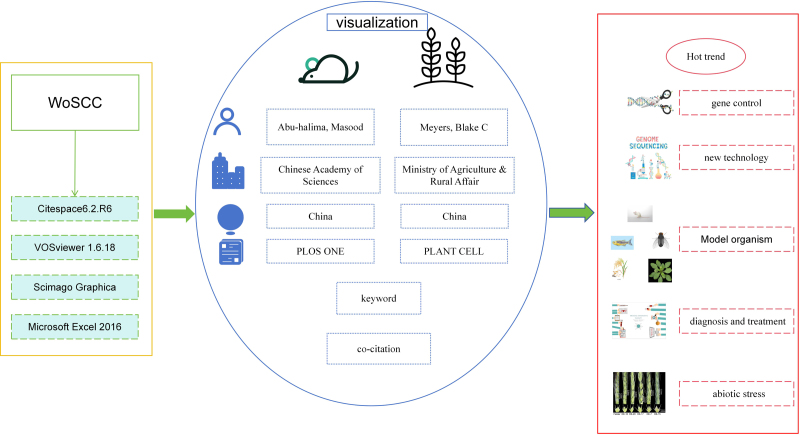

## 1. Introduction

Male infertility is a critically complex and concerning phenomenon in the vast biology field that affects both animals and plants by hindering reproduction and threatening species survival. While sometimes a result of the effect of external factors such as environmental pollution or diseases,^[1]^ male infertility predominantly arises due to internal genetic and physiological mechanisms. Because of advances in biotechnology and molecular biology, a new era of understanding mechanisms underlying gene expression regulation has emerged. Non-coding RNAs (ncRNAs) do not encode proteins but play pivotal roles in regulating gene expression. Research has revealed the key roles of ncRNAs in reproduction, stress responses, and disease states of animals and plants.^[2]^

Initially considered “junk,”^[3]^ accumulating evidence suggests that ncRNAs perform various regulatory activities in cells, including gene expression regulation, cellular regulation, and signaling.^[4,5]^ The transcription of a considerable amount of ncRNAs in animal and plant genomes is closely linked to their growth and development.^[6]^ Infertility in animals and plants affects biodiversity conservation and directly influences agricultural productivity and food safety. Infertility may be caused by factors such as sperm production problems, abnormal testicular development,^[7,8]^ and plant male sterility.^[9]^ Notable similarities are observed in ncRNA existence and functions across animals and plants. For example, the structure, function, and origins of long non-coding RNAs (lncRNAs) are highly similar in both plants and animals, which suggests regular patterns.^[10]^ Enhancing plant resilience and animal fertility through gene regulation is now a focal point of research.

However, despite advancements made in the study of ncRNAs, their specific roles and regulatory mechanisms in infertility remain to be completely elucidated. Bibliometrics, a scientific literature-analyzing method,^[11]^ assesses citation and publication trends to effectively reveal the knowledge structure and developmental dynamics within specific research areas. In the present study, bibliometric methods were used to systematically review and analyze literature on male infertility-related ncRNAs in animals and plants, so as to uncover research hotspots, key changes, and future trends. Few studies on ncRNAs in animal and plant male infertility were published in 2005. Therefore, we here selected literature published after 2005 for research. CiteSpace 6.2.R6 was used for the bibliometric analysis, along with Scimago Graphica, VOSviewer 1.6.18, and Microsoft Excel 2016, to explore research hotspots and trends in infertility-related ncRNAs in animals and plants since 2005 and to create visual knowledge maps. We aim to aid scholars in understanding the scientific foundation and research trends in this field, assessing hot issues, and exploring new findings in male infertility research by comparing similarities and differences in hotspots between animals and plants.

## 2. Materials and methods

### 2.1. Data collection

Web of Science (WoS), which is widely used in bibliometrics, is a comprehensive and authoritative database for global scholarly data.^[12]^ The Web of Science Core Collection (WoSCC) was primarily used. Data were retrieved from WoSCC on March 3, 2024. Covering publications from 2005 to 2023, we conducted a search using the following query: (ALL = (asthenospermia OR asthenozoospermia OR little weak sperm disease OR little weak essence disease OR less weak sperm syndrome OR cryptospermia OR oligozoospermia OR oligospermia OR teratozoospermia OR male infertility OR male sterility OR masculine sterility OR male barrenness OR male infertile OR infertile men OR infertile males)) AND ALL = (ncRNA OR miRNA OR long non-coding RNA OR non-protein coding RNA OR non-coding ribonucleic acid OR non-encoding RNAs OR lncRNA OR ceRNA OR piRNA OR miRNA). Only original articles and reviews were included in the present study. “Complete Record and Cited References” and “Plain Text” results were downloaded, and the documents were imported into NoteExpress for filtering. Data on animals and plants were analyzed separately by using CiteSpace software.

### 2.2. Data analysis

Three bibliometric tools, namely CiteSpace 6.2.R6, VOSviewer 1.6.18,^[13]^ and Scimago Graphica^[14]^ were used. CiteSpace was used to generate visual representations of countries/regions, institutions, journals, references, citations, and keywords through co-occurrence, timeline, burst detection. In the analysis, recommended procedures with a timeframe of 2005 to 2023 were followed by employing a yearly time slice, a selection criterion based on the g-index (*k* = 25), and pruning methods such as “pathfinder,” “pruning sliced networks,” and “pruning the merged network.”

VOSviewer, which can visualize knowledge maps, displays various clusters, overlays, or density colors.^[15]^ We primarily used the co-occurrence analysis function of VOSviewer along with Scimago Graphica to create geographical distribution maps of countries/regions. The network map presented the current state of research and exchange between these countries/regions.

The keywords with the same meaning and the names of countries belonging to the same region were merged and unified. For example, “long non-coding RNAs” and “lncRNAs” were merged into “lncRNA”; and “semen” and “sperm” were merged into “spermatozoa”; and “IRELAND” and “ENGLAND” were merged into “United Kingdom.”

To complement visualizations, Excel 2016 was used to dissect data collected using CiteSpace. Additionally, we accessed the latest impact factors from the WoS as of February 1, 2024.

## 3. Results

### 3.1. Annual growth trends from the WoSCC database

**Figure 1.**
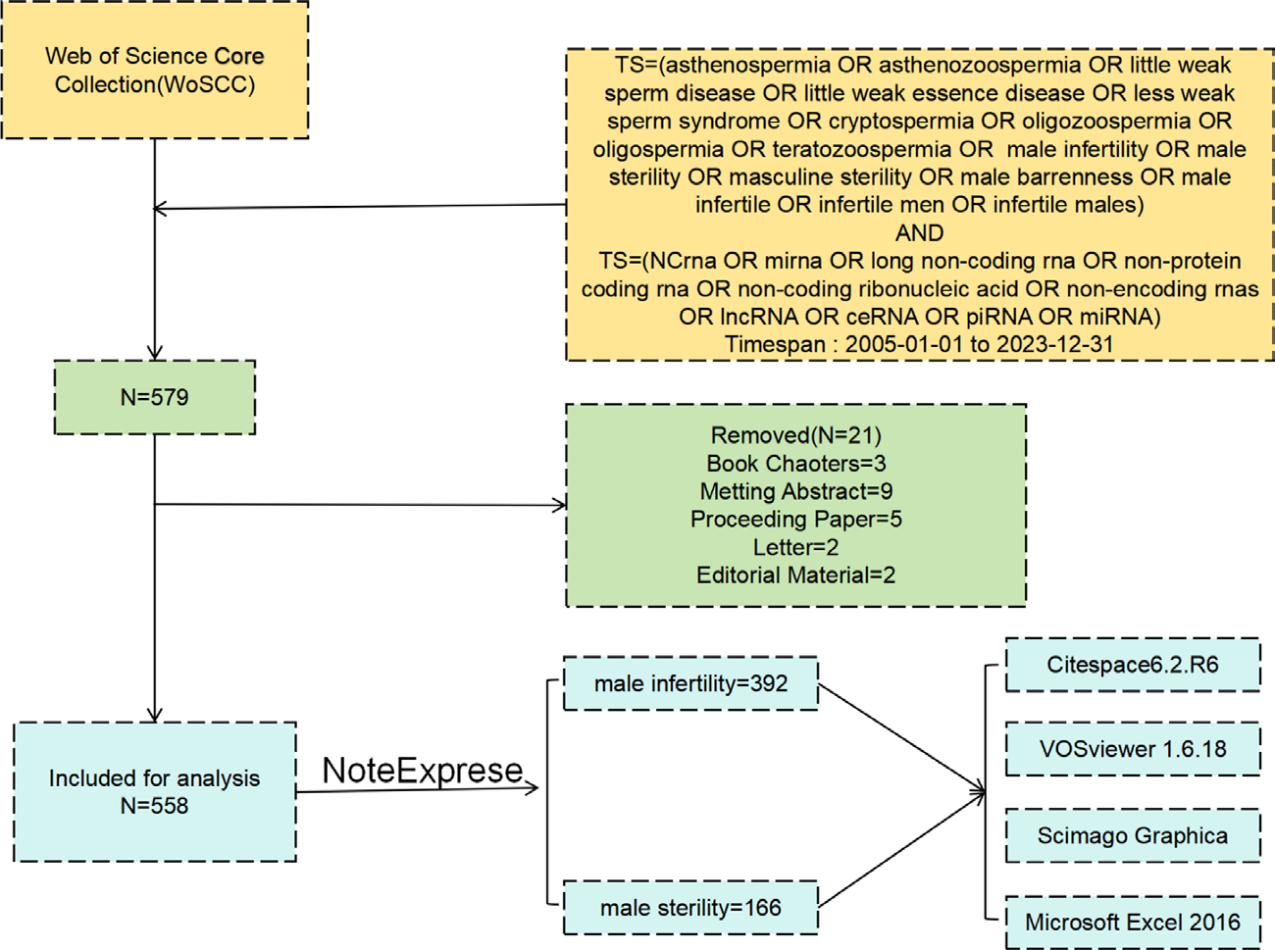
Flow chart of data screening and analysis.

**Figure 2.**
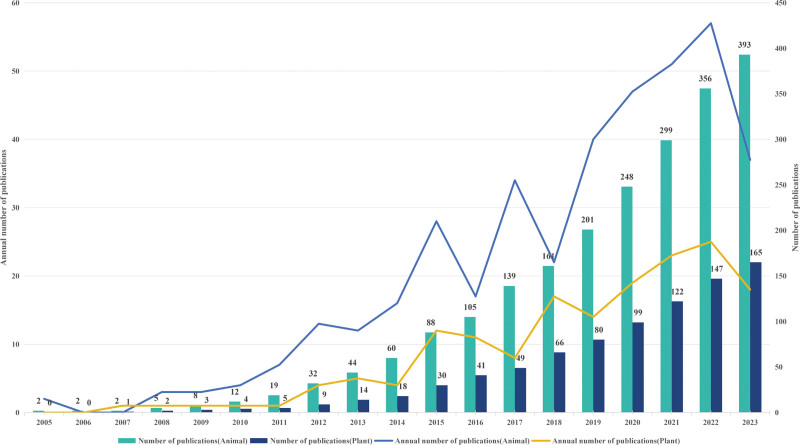
Annual release trend of non-coding RNAs in plants and animals from 2005 to 2023.

In total, 579 papers were retrieved from the WoSCC database. Of them, 558 papers met the inclusion criteria (Fig. 1). The screening process unveiled a fluctuating increase in the number of ncRNA and animal and plant male sterility-related publications. The growth in the number of articles related to ncRNAs in plants and animals has been steadily increasing on an annual basis. The number of articles on animals and plants, published annually, was the highest in 2022. The growth of articles on plant ncRNAs has been slower than that of articles on animal ncRNAs. A tendency toward slow growth was noted in 2023 possibly because all associated articles were not included (Fig. 2).

### 3.2. Distribution of countries/regions and institutions

**Figure 3.**
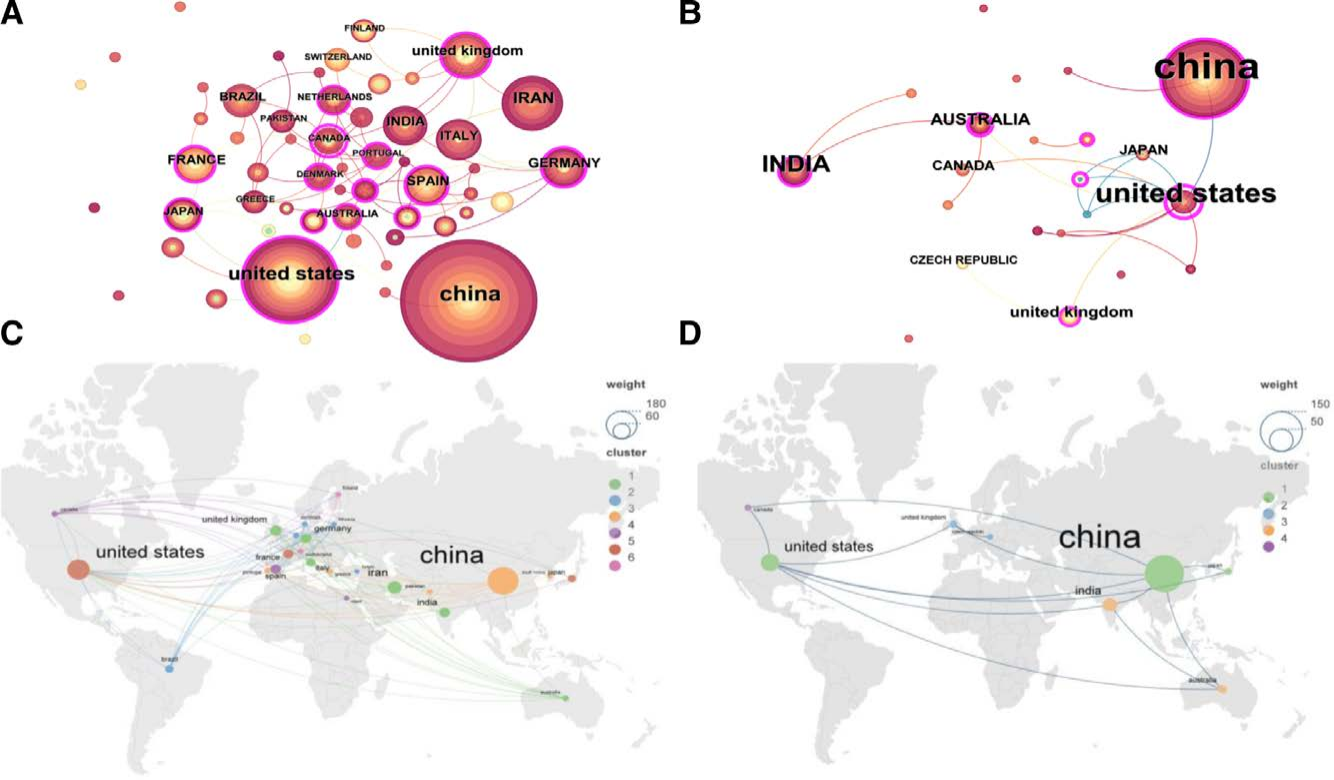
National visualization of non-coding RNAs in animal and plant studies.

Figure 3A and B is visualizations of animal and plant studies by countries, which were created using CiteSpace visualization software. In these figures, nodes represent research institutions, and the each node’s size corresponds to the co-occurrence frequency of the institutions. The diameter of each circle correlates with the number of papers that the institution has published, with a purple halo indicating greater centrality. In total, 392 articles on animal studies were contributed by 289 institutions across 57 countries. China and the United States had published the highest number of papers (Table 1), with 166 articles on plant studies from 23 different countries and 187 institutions. Only a smaller number of countries were involved in plant research, and China and the United States were again at the forefront in terms of publication volume. A network of country collaborations was established using VOSviewer and Scimago Graphica (Fig. 3C pertains to animals and Fig. 3D to plants). The network map presents the current state of research and communication activities in these countries/regions. The centrality values of China were 0.13 and 0.33 and lower than those of the United States (0.47 and 0.66, respectively). Plant research is relatively more abundant in China, with higher centrality.

**Table 1:** Top 14 countries publishing articles related to non-coding RNA research in animals and plants.

Rank	Countries/regions (animal)	Centrality	Count	Countries/regions (plant)	Centrality	Count
1	China	0.13	162	China	0.33	123
2	United States	0.47	87	United States	0.66	27
3	Iran	0.01	34	India	0.14	16
4	United Kingdom	0.14	23	Australia	0.03	6
5	India	0	20	United Kingdom	0.14	5
6	Germany	0.17	20	Canada	0	4
7	Spain	0.23	19	Japan	0.05	4
8	France	0.01	18	Czech Republic	0	3
9	Italy	0.02	16	France	0.26	2
10	Brazil	0.05	13	Denmark	0	2
11	Japan	0.01	12	Germany	0	2
12	Australia	0.07	8	Iran	0	2
13	Canada	0.11	7	Saudi Arabia	0.14	2
14	Netherlands	0.07	7	Spain	0	2

Fig. 3A and B presents visualizations of animal and plant studies by country. Figure 3C and D depicts the networks of country collaborations in animal and plant research.

Top 10 of the national institutions are depicted in Table 2. The Chinese Academy of Sciences topped the list of institutions for the number of papers published on animal research. The Ministry of Agriculture & Rural Affairs led in terms of volume of related to plant studies and exhibited a relatively high centrality.

**Table 2:** Top 10 institutions publishing articles related to non-coding RNA research in animals and plants.

Rank	Institution (animal)	Centrality	Count	Institution (plant)	Centrality	Count
1	Chinese Academy of Sciences (China)	0.12	19	Ministry of Agriculture & Rural Affairs (China)	0.16	19
2	Shanghai Jiao Tong University (China)	0.03	16	Chinese Academy of Agricultural Sciences (China)	0.03	15
3	Huazhong University of Science & Technology (China)	0.03	15	Huazhong Agricultural University (China)	0.03	13
4	University of California System (United States)	0.4	15	Chinese Academy of Sciences (China)	0.07	13
5	Centre National de la Recherche Scientifique (France)	0.07	12	Institute of Cotton Research (China)	0.01	8
6	Nanjing Medical University (China)	0.08	9	Beijing Academy of Agriculture & Forestry Sciences (China)	0.03	8
7	Cornell University (United States)	0.12	8	Institute of Genetics & Developmental Biology (China)	0.03	8
8	Chinese Academy of Agricultural Sciences (China)	0.1	8	China Agricultural University (China)	0.02	8
9	Academic Center for Education (Iran)	0.14	8	Indian Council of Agricultural Research (India)	0	7
10	Nanjing University (China)	0.03	8	Guangxi University (China)	0.04	5

### 3.3. Authors and co-cited authors

**Figure 4.**
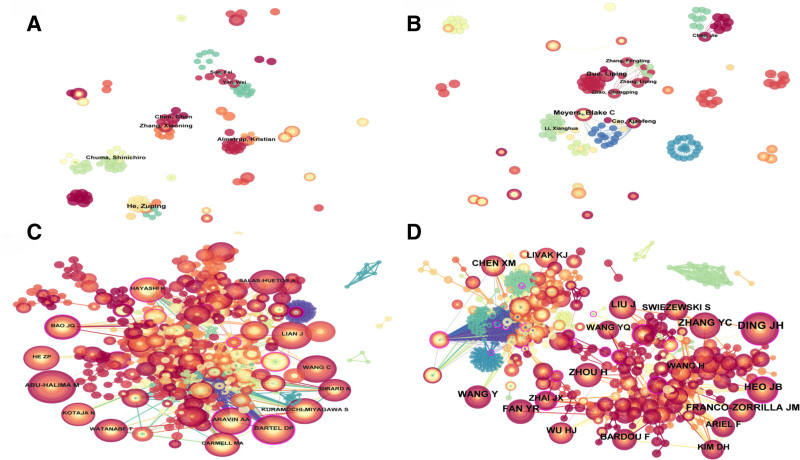
Author visualization of non-coding RNAs in animal and plant studies with author co-citation visualization.

By analyzing the author’s collaboration network, detailed scientific data can shared with other researchers, encompassing each author’s publication count, research focus, and collaborative ties. Regarding ncRNA research in animal male infertility, 421 authors actively contributed. The author visualizations are presented in Figure 4A. Regarding ncRNA research in plant male infertility, 341 authors were notably active, with their visualizations presented in Figure 4B. Table 3 presents the top 10 authors publishing about ncRNA in animal and plant studies, along with their co-cited authors. The highest publication output in animal studies was noted for Abu-Halima, Masood, and that in plant studies was noted for Meyers, Blake C.

**Table 3:** Top 10 authors publishing about non-coding RNAs in animal and plant studies are co-cited with authors.

Rank	Author (animal)	Count	Co-cited author	Count	Author (plant)	Count	Co-cited author	Count
1	Abu-halima, Masood	7	Abu-Halima M	94	Meyers, Blake C	6	Ding JH	86
2	He, Zuping	5	Bartel DP	75	Guo, Liping	5	Zhang YC	59
3	Blanco, Joan	5	Aravin AA	68	Cao, Xiaofeng	4	Heo JB	53
4	Zhao, Wangsheng	5	Lian J	68	Chen, Yue-Qin	4	Zhou H	50
5	Anton, Ester	4	Wang C	66	Ding, Yi	4	Franco-Zorrilla JM	48
6	Chen, Chen	4	Kuramochi-Miyagawa S	60	Cao, Jiashu	4	Liu J	48
7	Almstrup, Kristian	4	Salas-Huetos A	53	Zhang, Fengting	3	Fan YR	47
8	Liu, Lin	4	Hayashi K	53	Guo, Wen-Wu	3	Wang Y	44
9	Chuma, Shinichiro	4	Kotaja N	52	Li, Xianghua	3	Chen XM	38
10	Zhang, Xiaoning	4	Watanabe T	51	Gai, Junyi	3	Bardou F	37

Figure 4A and B presents visualizations of animal and plant studies by authors. Figure 4C and D presents visualizations of animal and plant studies by author co-citations.

Active partnerships were observed in animal author visualizations, presenting Chen, Chen, Zhang, Xiaoming, Sun, Fei, and Yan, Wei in a common cluster. In plant author visualization, diverse authors such as Meyers, Blake C, Cao, Xiaofeng, and Li, Xianghua were grouped in a cluster.

Figure 4C and D presents the co-cited author visualizations for animals and plants. In animal research, Abu-Halima, Masood was identified as the most frequently co-cited author (Fig. 4C and Table 3). DING JH was identified as the most co-cited author in plant research (Fig. 4D and Table 3).

### 3.4. Journals and co-cited academic journals

Table 4 presents the most frequently cited journals in the research field of male infertility-related ncRNAs. The table depicts the number of most frequently cited journals and the journal impact factor in 2023. *PLoS ONE* is the most co-cited journal for animal studies, and *The Plant Cell* is the most co-cited journal for plant studies.

**Table 4:** Top 10 co-cited journals for research on non-coding RNAs in animals and plants.

Rank	Co-cited Journal (animal)	Count	Impact factors (2023)	Co-cited journal (plant)	Count	Impact factors (2023)
1	*PLoS One*	271	2.9	*Plant Cell*	155	10.0
2	*Cell*	255	45.6	*Plant Journal*	149	6.2
3	*P Natl Acad Sci USA*	252	9.4	*P Natl Acad Sci USA*	147	9.4
4	*Nature*	247	50.5	*Plant Physiology*	142	6.6
5	*Biol Reprod*	247	3.1	*Nucleic Acids Res*	142	16.7
6	*Nucleic Acids Res*	219	16.7	*Cell*	136	45.6
7	*Fertil Steril*	208	6.6	*Science*	125	44.8
8	*Science*	203	44.8	*BMC Genomics*	124	3.5
9	*Reproduction*	201	3.7	*Genome Biology*	119	10.1
10	*Hum Reprod*	198	6.0	*BMC Plant Biol*	119	4.3

### 3.5. Co-cited references and reference bursts

**Figure 5.**
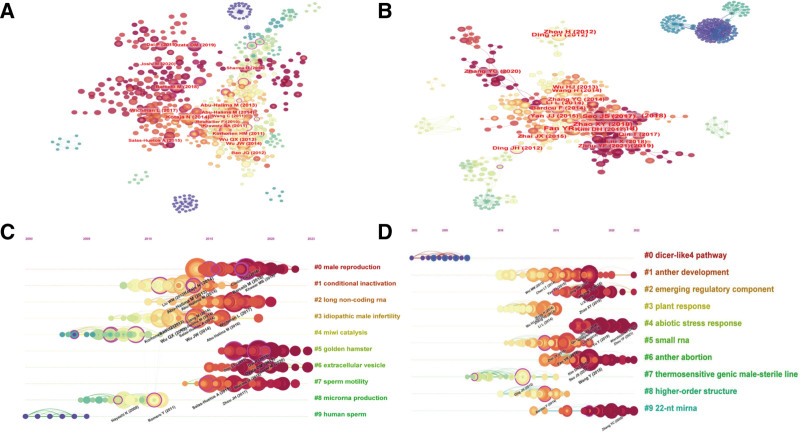
Visualization of co-citation of non-coding RNAs in animal and plant studies and a time plot with (title) as the cluster.

Table 5 presents the top 10 most frequently cited references of non-coding RNAs in animal and plant studies, with the lowest co-citation frequency recorded at 103 times. The selected articles were visualized as co-cited and organized using clustering functions, which potentially divided the entire network map into several clusters. Subsequently, a corresponding timeline chart was generated (Fig. 5). Figure 5A and B is visualizations of animal and plant studies by co-citation. In these figures, the nodes represent individual co-citations, with the size of each node corresponding to the frequency of their co-occurrence in the literature. The diameter of each circle is proportional to the number of papers published by the co-citation, with a purple halo indicating higher centrality within the network. In animal research, the most cited article is of N Kotaja, which was published in *Fertility and Sterility* in 2014 (n = 21).^[16]^ In plant research, the most cited article is of Wang Y. et al, which was published in *Nature Communications* in 2018 (n = 26).^[17]^ Additionally, among the top 10 most cited articles on animal studies, 2 were review articles. Among of the top 10 most cited articles on plant studies, 1 was a review article.

**Table 5:** Top 10 co-cited references of non-coding RNAs in animal and plant studies.

Rank	Title (animal)	Citations	Type	Year	Title (plant)	Citations	Type	Year
1	Kotaja N, 2014, *Fertil Steril*, V101, P1552, DOI 10.1016/j.fertnstert.2014.04.025^[16]^	289	Reviews	2014	Wang Y, 2018, *Nat Commun*, V9, P0, DOI 10.1038/s41467-018-05829-7^[17]^	184	Article	2018
2	Abu-Halima M, 2013, *Fertil Steril*, V99, P1249, DOI10.1016/j.fertnstert.2012.11.054^[18]^	274	Article	2013	Fan YR, 2016, *P Natl Acad Sci USA*, V113, P15144, DOI10.1073/pnas.1619159114^[19]^	245	Article	2016
3	Wu QX, 2012, *J Biol Chem*, V287, P25173, DOI 10.1074/jbc.M112.362053^[20]^	226	Article	2018	Huang L, 2018, *Plant J*, V96, P203, DOI 10.1111/tpj.14016^[21]^	104	Article	2018
4	Wichman L, 2017, *Biol Reprod*, V97, P313, DOI 10.1093/biolre/iox084^[22]^	101	Article	2017	Zhao XY, 2018, *Nat Commun*, V9, P0, DOI 10.1038/s41467-018-07500-7^[23]^	267	Article	2018
5	Ozata DM, 2019, *Nat Rev Genet*, V20, P89, DOI 10.1038/s41576-018-0073-3^[24]^	923	Article	2019	Ding JH, 2012, *P Natl Acad Sci USA*, V109, P2654, DOI 10.1073/pnas.1121374109^[25]^	637	Article	2012
6	Wu JW, 2014, *P Natl Acad Sci USA*, V111, PE2851, DOI 10.1073/pnas.1407777111^[26]^	281	Article	2014	Seo JS, 2017, *Plant Cell*, V29, P1024, DOI 10.1105/tpc.16.00886^[27]^	196	Article	2017
7	Barceló M, 2018, *Hum Reprod*, V33, P1087, DOI 10.1093/humrep/dey072^[28]^	144	Article	2018	Zhou H, 2012, *Cell Res*, V22, P649, DOI 10.1038/cr.2012.28^[29]^	334	rtical	2012
8	Abu-Halima M, 2014, *Fertil Steril*, V101, P78, DOI 10.1016/j.fertnstert.2013.09.009^[30]^	166	Reviews	2014	Yu Y, 2019, *Annu Rev Cell Dev Biol*, V35, P407, DOI 10.1146/annurev-cellbio-100818-125218^[31]^	252	Reviews	2019
9	Dai P, 2019, *Cell*, V179, P1566, DOI 10.1016/j.cell.2019.11.022^[32]^	149	Article	2019	Li L, 2014, *Genome Biol*, V15, P0, DOI 10.1186/gb-2014-15-2-r40^[33]^	462	Article	2014
10	Korhonen HM, 2011, *PLoS One*, V6, P0, DOI 10.1371/journal.pone.0024821^[34]^	184	Article	2011	Yan JJ, 2015, *Planta*, V241, P109, DOI 10.1007/s00425-014-2167-2^[35]^	58	Article	2015

The timeline view of references allows users to determine how research hotspots have evolved over time. Each of these clusters may focus on different research themes, clustered by “T (title).” As shown in Figure 5C and D, clusters #9 (human sperm) and #0 (dicer-like4 pathway) started early but also concluded early. Meanwhile, animal clusters #5 (golden hamster) and #6 (extracellular), and plant clusters #1 (anther development), #2 (emerging regulatory component), #4 (abiotic stress response), #6 (anther abortion), and #9 (22-nt miRNA) were still active, which can be considered as cutting-edge. According to the charts, animal T clusters mainly focused on themes such as male reproduction, sperm motility, and human sperm quality. These themes involve biological processes such as gene expression, protein synthesis, and microRNA (miRNA) production, with a particular emphasis on reproductive health issues including idiopathic male infertility and non-obstructive azoospermia (NOA). The plant T clusters were dedicated to aspects of plant reproduction, especially topics such as another development, flowering timing, and cytoplasmic male sterility. Moreover, they involved discussions on topics such as abiotic stress, plant responses, and environmental shifts within ecology and environmental science.

Figure 5A and B is visualizations of animal and plant studies by co-citation. Figure 5C and D illustrates the time plots of animal and plant studies, with the title representing the cluster.

**Figure 6.**
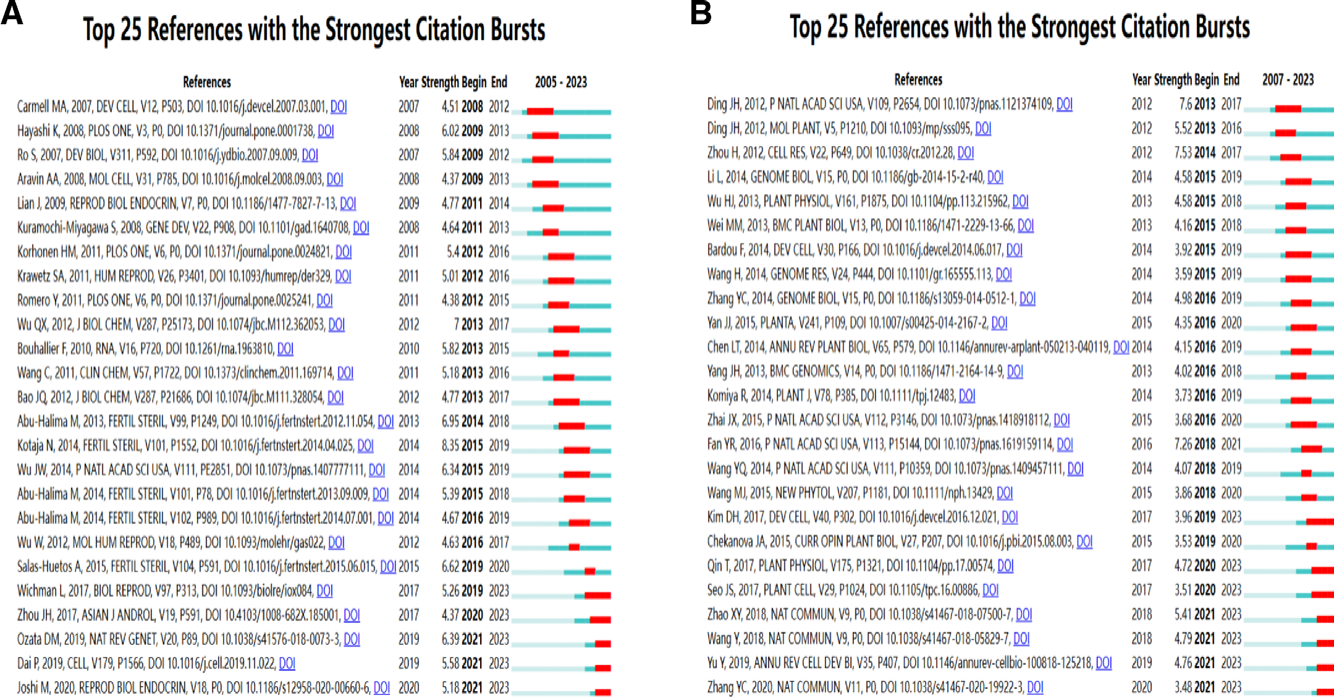
Top 25 co-cited bursts of non-coding RNAs in animal and plant studies. (A) Animal co-cited bursts; (B) plant co-cited bursts.

The citation burst analysis underscores the top 25 most cited references (Fig. 6A and B). A significant burst was observed for N Kotaja et al’s article “MicroRNAs and spermatogenesis,” published in *Fertility and Sterility* in 2010, with the highest burst strength (8.35) observed between 2015 and 2019.^[16]^ Among plant studies, Ding paper “A long noncoding RNA regulates photoperiod-sensitive male sterility, an essential component of hybrid rice,” published in 2012 in *Proceedings of the National Academy of Sciences*, had the highest citation burst strength (7.49) during 2008 to 2012.^[25]^ Remarkably, 4 animal study-related papers and 5 plant stud-related papers remain in their citation burst phase.

### 3.6. Study of trends in keyword analysis

**Figure 7.**
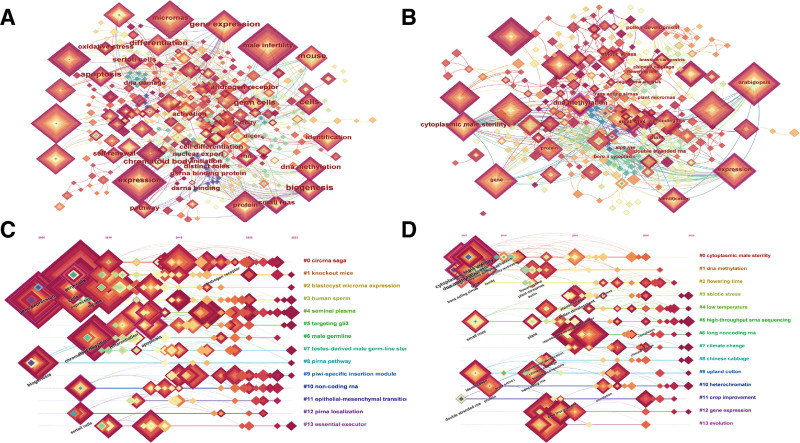
Visualization of keywords related to non-coding RNAs in animal and plant studies and a time plot with (keyword) as the cluster.

The visualization of keywords, keyword clustering, and timeline view provides invaluable insights into current trends and future directions in animal and plant reproductive research (Fig. 7). Figure 7A and B is visualizations of animal and plant studies by keyword. In these figures, the nodes represent individual keywords, with the size of each node corresponding to the frequency of their keywords in the literature. The diameter of each circle is proportional to the number of papers published by the keywords, with a purple halo indicating higher centrality within the network. The 12 leading keywords are presented in Table 3. The keywords most frequently used in animal and plant studies are “male infertility” and “Arabidopsis,” and those with the highest centrality are “expression” and “cytoplasmic male sterility.” In animal and plant studies, “gene expression” ranks high as a keyword and has significant centrality, which indicates that gene regulation is a focal point in infertility research in animals and plants. Although “lncRNA” is infrequently used in plant research, its high centrality highlights its increasing significance within this field. These analyses unveil that interdisciplinary research approaches can allow knowledge exchange and technological innovation between the 2 fields. The prevalent occurrence and centrality of genes and ncRNAs in both fields underscore the centrality of gene regulatory mechanisms in biological studies. Considering the prominent centrality of lncRNA, lncRNA is a burgeoning area of focus in plant research. This research area is likely to attract additional studies on its functions and regulatory mechanisms. In both animal and plant research, model organisms such as “mouse” and “golden hamster,” and “Arabidopsis,” respectively are instrumental in elucidating complex biological processes and fostering developments in disease diagnosis^[36]^ and genetics.^[37]^ The rankings and centrality of these keywords help comprehend current research focal points and potential areas for future development. Future studies may focus on these areas, thereby employing novel technologies and methodologies for propelling scientific advancements.

Using “K (keyword)” for clustering, the co-occurrence and clustering of animal and plant keywords were visualized, each yielding 13 clusters. Subsequently, the corresponding timeline graphs was generated. The timeline graphs for animals and plants indicate that 9 of the 13 animal clusters were active, with 9 plant clusters also being active (Fig. 7C and D). Clusters #3 and #8 in animals started the earliest, and clusters #0, #2, and #11 were the earliest in plants. Animal research clusters focused more on Circular RNAs (circRNAs) and Piwi-interacting RNAs (piRNAs), all ncRNAs related to miRNAs, predominantly used in disease diagnosis and treatment, with specifically emphasizing on clinical applications (Table 6). Plant clusters focused more on lncRNAs, emphasizing on specific techniques such as DNA methylation, high-throughput sequencing, and whole-genome identification. This indicated that these technologies are critical for plant genetics research. Model organisms used in both animal and plant studies underscore the significance of model systems in ncRNA research.

**Table 6:** Top 12 keywords of non-coding RNAs in animal and plant studies.

Rank	Keyword (Animal)	Count	Centrality	Keyword (Plant)	Count	Centrality
1	Male infertility	133	0.15	Arabidopsis	68	0.16
2	Expression	104	0.24	Sensitive male sterility	54	0.08
3	Spermatogenesis	93	0.17	Male infertility	53	0.06
4	MicroRNAs	82	0.08	Expression	49	0.2
5	Spermatozoa	68	0.09	Gene	48	0.21
6	Protein	64	0.09	MicroRNAs	48	0.07
7	Gene	62	0.07	lncRNA	39	0.08
8	Gene expression	53	0.13	Cytoplasmic male sterility	33	0.27
9	Messenger RNA	37	0.08	Messenger RNA	32	0.06
10	Mouse	37	0.14	Identification	28	0.08
11	DNA methylation	36	0.06	Gene expression	28	0.07
12	lncRNA	36	0.05	Antisense transcripts	24	0.09

Figure 7A and B is visualizations of animal and plant studies by keyword. Figure 7C and D depicts time plots of animal and plant studies, with keyword serving as the cluster.

**Figure 8.**
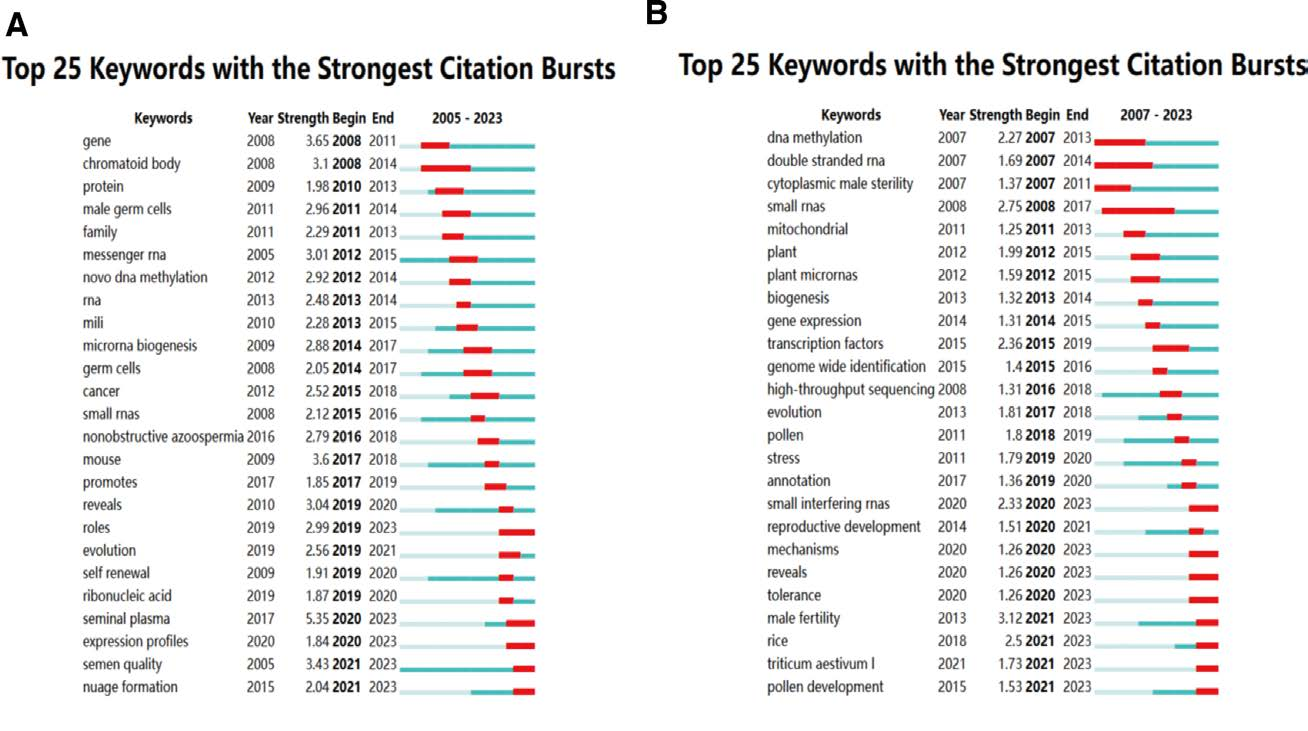
Top 25 keyword bursts of non-coding RNAs in animal and plant studies. (A) Animal keyword bursts; (B) plant keyword bursts.

The burst analysis of keywords has revealed marked increases in certain animal- and plant reproduction-related terms, which indicates focal points of current research. According to Figure 8A and B, in animal research, “seminal plasma” had the highest burst intensity from 2020 to 2023 (5.35), and in plant research, “male fertility” peaked from 2021 to 2023 (3.12). Topics remaining in the burst status by 2023 include “roles,” “seminal plasma,” “expression profiles,” “semen quality,” “nuage formation,” “small interfering RNAs,” “mechanisms,” “reveals,” “tolerance,” “male fertility,” “rice,” “Triticum aestivum L,” and “pollen development.” Overall, keyword emergence in animal and plant studies suggests that genes and their expression, ncRNAs and their production, reproductive cells and health, research methodologies and technologies, model organisms and specific diseases, and emerging research areas will be focused in future studies.

## 4. Discussion

### 4.1. General information

We here comprehensively analyzed academic publications on male infertility-related ncRNAs in animals and plants, thereby covering multidimensional contributions of journals, countries, research institutions, and authors. By using bibliometric methods to extensively analyze these highly cited papers, research hotspots, development changes, and future trends in this field were revealed, thereby generating a series of insightful conclusions. According to us, this study is the first to use the WoS database for bibliometric analysis and knowledge mapping of research about male infertility-related ncRNAs in animals and plants. We noted that although the number of papers published in animal and plant fields unveiled a slow and fluctuating upward trend, the number and growth rate of animal studies were higher than those of plant studies, which reflected that higher academic attention has been paid to male infertility in animals. This phenomenon may be closely related to the widespread application and in-depth exploration of ncRNA research in the medical field.

The number of published articles is a crucial indicator,^[38]^ but centrality is a vital indicator for measuring article quality in the national/region analysis.^[39]^ High centrality nodes (≥0.10) indicate the “hub” influence in the global cooperation map.^[40]^ Regarding publishing countries, China ranks first in terms of volume of publications in both animal and plant fields. However, its centrality is lower than that of the United States, particularly in animal research. China’s centrality is relatively higher, which indicates China’s leading position in agricultural research worldwide. The United States, Spain, Germany, Portugal, the United Kingdom, and China have higher centrality in animal research. Canada, the United States, China, France, India, the United Kingdom, and Saudi Arabia have higher centrality in plant research, which indicated that they are vital hubs for international cooperation in ncRNA and infertility research. China ranks first in terms of publishing institutions, especially in plant research, with 9 of the top 10 institutions being Chinese. However, Chinese institutions have lower centrality. Regarding centrality, the University of California System in the United States ranks first in animal research, followed by the Academic Center for Education in India, and Monash University in Australia despite publishing only 2 articles. The Chinese Academy of Sciences ranks fourth in animal research. This indicates that Chinese institutions predominantly collaborate with domestic institutions, especially in plant research. By contrast, American institutions engage more in international exchanges, which shows that academic exchanges on the international stage are limited in China.

Professor Abu-Halima is the most published and cited scholar in animal research, and his research base is Saarland University, Germany. He and his team have significantly contributed to the fields of male infertility and ncRNAs, particularly miRNAs. Their research has extensively focused on the roles of miRNAs in sperm production, activity, and semen expression, thereby amply exploring the expression profiles and regulatory effects of miRNAs on reproductive cells. From 2013 to 2023, the team published numerous crucial articles revealing differences in miRNA expression profiles between normal and dysfunctional sperm (such as oligospermia and asthenospermia). This suggests that miRNAs can be used as diagnostic tools for male infertility.^[18]^ They also investigated the regulatory role of miRNAs in male reproductive and somatic cells and their association with reproductive abnormalities,^[30]^ and miRNA expression in extracellular vesicles in the seminal plasma of men with oligoasthenozoospermia.^[41]^ Additionally, they used microarray technology and real-time quantitative PCR combined with bioinformatics analysis they analyzed the miRNA expression profiles of fertile men and men with obstructive azoospermia.^[42]^ Professor Abu-Halima research has expanded our understanding of molecular mechanisms underlying male infertility and offered a scientific basis for developing miRNA-based new diagnostic tools and treatment strategies. Their work is significantly influential in the male reproductive health field and paves new directions for clinical applications.

Professor Meyers, from the University of California, Davis, has made substantial achievements in the plant ncRNA field, particularly while studying Phased small interfering RNAs (phasiRNAs), lncRNAs, and miRNAs. In 2008, Professor Meyers and his team discovered that miRNAs act by cleaving Messenger RNAs (mRNAs), which clarified the importance of the Dicer-like 1 enzyme in miRNA maturation.^[43]^ In 2013, they revealed the intricate interactions occurring between miRNAs and various genetic elements, and thus examined their evolutionary paths.^[44]^ Their 2016 research focused on the role of phasiRNAs in plant development.^[19]^ They have recently conducted in-depth studies on ncRNA functions in plant reproduction, particularly in regulating reproductive barriers in certain plants.^[45–47]^ Notably, Professor Meyers’ lab was among the first to study small RNAs by using high-throughput sequencing technology, thereby developing various bioinformatics tools that substantially contributed to the understanding of small RNA functions and mechanisms. Their work has advanced our knowledge about plant gene regulatory mechanisms and provided their potential applications for crop improvement and agricultural production. Professor DING JH from Huazhong Agricultural University is among the most cited researchers in the plant research field. He is particularly known for his breakthroughs in the study of rice nuclear sterility genes, which gave vital evidence for functional research on ncRNAs.^[25]^

In bibliometrics, the journal *PLoS ONE* excels in the ncRNA research field in animals because of its open access policy. It has published numerous articles on sperm maturation, embryonic development, the nervous system, and cancer research, thereby significantly advancing science in these areas.^[48]^ The journal *The Plant Cell* has also immensely influenced the plant research field, being among the journals with a high citation rate. This journal publishes research covering various roles of ncRNAs in plant growth, stress responses, biotic stress, plant reproduction, and transcriptional regulation, which enhances the understanding of their roles in plant physiology and allows to develop and implement new strategies for plant genetic improvement and agricultural production.^[49–51]^ Top journals such as *The Cell* and *Science* also have high citation frequencies in animal and plant research fields. The high h-index and impact factors of these journals indicate the extensive influence of the high-quality, comprehensive research they publish. The performance of other highly cited journals also confirms the vital role of high-impact factor journals in augmenting scientific research impact.

Knowledge networks constructed by analyzing co-cited academic papers reveal the mutual influence of academic fields. Of note, in animal research, the review article “MicroRNAs and spermatogenesis” is distinguished by its high citation rate and rapid increase in citations within the scholarly community. This article extensively discusses the critical regulatory roles of miRNAs in sex chromosomes and meiosis, and their role as biomarkers for assessing male sperm quality, thus offering novel diagnostic perspectives for male fertility.^[16]^ The analyzed top 10 co-cited articles majorly focused on miRNAs, particularly their role in mammalian spermatogenesis and male fertility. Moreover, 3 articles^[22,24,32]^ remain in a citation burst phase, which emphasizes the influence of piRNA, lncRNA, and Dicer on spermatogenesis and reproductive development. Dicer play crucial roles in the biogenesis of miRNAs. Research has found that a deficiency of Dicer in the male germ cell lineage leads to insufficient production of miRNAs and male infertility. This finding highlights the significance of these enzymes not only in miRNA processing but also in maintaining reproductive health, potentially paving the way for novel therapeutic approaches to treat infertility issues related to miRNA dysregulation.^[20]^ Several papers have also explored the role of lncRNAs, particularly the review article “Long non-coding RNAs (lncRNAs) in Spermatogenesis and Male Infertility,” which highlights that the role of lncRNAs in spermatogenesis is not yet completely understood.^[52]^ These studies have not only advanced our knowledge of the molecular aspects of spermatogenesis but also opened up new avenues for developing new diagnostics and treatments for male infertility.

The role of lncRNAs has increasingly garnered immense attention, particularly in the areas of plant reproductive development and crop improvement. Articles^[17,21,23,25,27,33]^ have focused on the crucial role of lncRNAs in these processes, thereby demonstrating the mechanisms underlying lncRNA-mediated regulation of gene expression through interactions with specific proteins or miRNAs. These articles have thus provided detailed insights into the diversity and importance of lncRNAs in plant biology. These studies have also highlighted the role of lncRNAs in plant male sterility, developmental regulation, immune responses, and environmental adaptability. Of the top 10 articles, 4 are still in the burst phase.^[17,23,27,31]^ These studies have explored the gene expression regulation and biosynthesis mechanisms of lncRNAs in *Arabidopsis* and highlighted their crucial role in stress responses. The findings suggested that lncRNAs enable plants to adapt to environmental changes, which is remarkably important for future plant science and agricultural production.

### 4.2. Hotspots and trends

By analyzing keywords, keyword clustering, emerging keywords, and co-citation analysis, current research hotspots and trends can be identified, which primarily include gene regulation, application of new technologies, biological models, diagnostics and treatment in animals, and crop improvement and abiotic stress in plants.

#### 4.2.1. Gene control

Gene regulation is a key mechanism underlying the study of ncRNA’s role in infertility. ncRNAs are known to be a pivotal player in the genetic regulation of spermatogenesis and male infertility.^[53]^ miRNAs regulate key genes involved in spermatogenesis and are closely associated with conditions such as NOA. They are thus crucial for male reproductive cell function and overall reproductive health.^[54,55]^ For instance, the anti-Müllerian hormone may serve as a biomarker for semen quality in dogs, which highlights the significance of hormonal regulation and ncRNA interactions in reproductive health.^[56]^ Moreover, the cysteine-rich secretory protein is a significant player in inflammatory responses and male infertility, and studies have shown that miR-182-5p, miR-192-5p, and miR-493-5p are negatively correlated with cysteine-rich secretory protein, thus regulating male infertility.^[57]^

Adenosine deaminase domain 2 is a testis-specific protein critical for reproduction in male mice. Adenosine deaminase domain 2 depletion reduces the level of the cluster-derived coarse filament piRNA and increases the expression of ping-pong-origin piRNA, which are both directly and indirectly involved in piRNA production.^[58]^

The interactions among ncRNAs also prove their importance. For instance, longer piRNAs have additional complementarity with target mRNAs, which thus facilitates translation activation and spermatocyte development.^[59]^ Mutation of protein poly(A)-specific RNase-like domain containing 1, a necessary piRNA modifier in the mouse lineage, leads to spermatogenesis failure and azoospermia.^[60]^ Acetylcholinesterase levels are notably higher in infertile men than in fertile men, indicating that the miRNA network regulates the cholinergic signaling pathway.^[61,62]^ Studies have recently uncovered the role of circRNAs in spermatogenesis; they are potentially involved in sperm production failure, sperm defects, and male infertility.^[63]^

However, research on phasiRNAs, lncRNAs, and circRNAs in plants is still in its early stages, and many related issues are yet to be resolved. In plants, ncRNAs are pivotal players in gene regulation under adverse conditions; they indirectly affect processes such as pollen development and male reproduction.^[64]^ Specific transcription factors, such as the basic Helix-Loop-Helix family, regulate stress tolerance-related genes, further linking ncRNAs to gene regulatory networks essential for plant reproduction.^[65]^ DNA methylation is a crucial epigenetic modification often studied alongside RNA- and ncRNA-mediated gene silencing. It is a key player in processes such as embryonic development, X-chromosome inactivation, genomic imprinting, and cell differentiation. Furthermore, DNA methylation and ncRNAs have a close interaction, potentially involving the integration of epigenetic transgenerational inheritance, and this highlights the major role of DNA methylation in genetics.^[66]^ In both animals and plants, aberrant DNA methylation patterns are linked to various forms of infertility, and this demonstrates the critical role of DNA methylation in their genetics.^[67]^ These studies have emphasized the role of DNA methylation and ncRNAs in gene regulation and their potential applications in infertility treatment, offering new directions and challenges for future research.

#### 4.2.2. Application of new technology

Advanced technologies such as high-throughput sequencing have recently enabled researchers to extensively investigate the role of ncRNAs in infertility in both animals and plants. These technologies have substantially increased the research efficiency and unveiled the critical role of ncRNAs in reproductive mechanisms. For instance, RNA sequencing (RNA-seq) technology precisely measures the expression levels of various ncRNAs, thereby unveiling functions of these ncRNAs in male infertility mechanisms. In studies on male sterility in plants, small RNAs related to infertility and their target genes were identified through RNA-seq data analysis.^[68]^ Single-cell RNA sequencing (scRNA-seq) analyzes ncRNA expression at the single-cell level and thus reveals cellular heterogeneity and its role in male infertility.^[69]^ For example, scRNA-seq is used the investigate changes in ncRNA expression in reproductive cells at various developmental stages, thereby exploring its regulatory mechanisms in infertility in animals and plants. Moreover, techniques, such as whole-genome small RNA-seq, transcriptome sequencing, degradome sequencing, and short tandem target mimic, have been used to develop transgenic plants to further identify fertility-associated miRNAs and establish regulatory networks.^[35,70,71]^ Through small RNA-seq, Oladele A. Oluwayiose identified various ncRNAs in seminal extracellular vesicles and noted alterations in the expression of 57 ncRNAs in men with subpar sperm quality, of which 60% was circRNAs. Functional analyses have revealed that the target genes of differentially expressed miRNAs and circRNAs are associated with cell communication- and early development-related functions.^[72]^ Edmundo Domínguez-Rosas and colleagues suggested that new technologies allow researchers to achieve a deeper understanding of lncRNA-related biological functions and regulatory mechanisms, unveiling the molecular mechanisms underlying plant growth and response to environmental stress.^[73]^ In interspecies studies, Liesbeth Minnoye and her team developed and validated a deep learning model known as DeepMEL. This model combines epigenomics with deep learning to provide a framework for analyzing the logic underlying melanoma enhancers, which are suitable for specific cell types or conditions.^[74]^

In addition to high-throughput sequencing and gene editing techniques, functional experiments, such as overexpression and knockout studies, are vital for studying ncRNA functions, thereby paving the way for the treatment of human infertility.^[75]^ A comprehensive study of male infertility can be conducted by combining genomics, transcriptomics, proteomics, and metabolomics data. This integrative analytical method allows a comprehensive understanding of the role of ncRNAs in reproductive health and assists in identifying new biological markers.

#### 4.2.3. Biological model

The utilization of ncRNAs for studying animal and plant male infertility by employing various biological models has recently made remarkable progress.^[76]^ Some common models and their applications in ncRNA studies have been listed below:

Mouse model: Mouse is a model commonly used for investigating ncRNAs in male infertility.^[77]^ Through gene knockout and transgenic technologies, researchers can extensively explore the role of ncRNAs in spermatogenesis, sperm morphology, quantity, differentiation, and so on.^[34]^

Model: Zebrafish is an ideal model for studying reproductive biology and gene functions because it has a rapid developmental cycle and transparent embryos. A specific ncRNA played crucial roles in germ cell development and infertility in zebrafish.^[78]^ Zebrafish was used to investigate the role of a specific ncRNA in germ cell development. Using Clustered Regularly Interspaced Short Palindromic Repeats-Associated Protein 12a for gene knockout and rescue experiments, the function of ncRNAs in cell proliferation and development was explored.^[79]^

*Drosophila*: As a classic genetic model, *Drosophila* is used to determine the regulatory mechanism of ncRNA in reproductive development and stress response.^[80]^ Various stages of the life cycle are accompanied by hormonal fluctuations and changes in the metabolic system, which provides a platform for studying the function of small RNAs in germ cell development. LncRNA expression patterns in the *Drosophila* ovary and testis were explored, and the understanding of the role of ncRNAs in germ cell maturation and function was enhanced.^[81]^

*Arabidopsis*: As a standard model of plant biology, the simple morphological structure and rapid life cycle of *Arabidopsis* make it an ideal model for exploring gene function. *Arabidopsis* plays a key role in the study of ncRNA function, particularly while investigating gene silencing mechanisms and their role in plant growth and stress response.^[82]^

Rice: As a major crop, rice is used to investigate the regulatory role of ncRNAs in pollen development and male sterility, especially the reproductive response in response to environmental stress.^[17]^

Using these biological models, together with advanced gene editing and sequencing technologies, has allowed scientists to achieve a deeper understanding of the complex role played by ncRNA in the sterility mechanism, thereby offering the basis for developing potential therapeutic strategies.

#### 4.2.4. Animals in the diagnosis and treatment

Male infertility is a global problem affecting more than 5 million couples. It includes impaired spermatogenesis, abnormal testicular development, and sperm maturation problems. Although conventional diagnostic methods such as semen analysis, karyotyping, and Y chromosome microdeletion assessment are somewhat useful, these methods are expensive and time-consuming, and produce uncertain results. NcRNA has recently been considered an ideal biomarker because of its specific expression in reproductive tissues and its high stability, sensitivity, and non-invasiveness.^[83]^ In particular, it is useful for detecting a specific miRNA in human semen^[84]^ and has the potential to assess male fertility.^[28]^ Additionally, ncRNAs have been implicated in most human diseases, ranging from neurological diseases to cardiovascular diseases.^[6]^ They have also exhibited potential in the therapeutic field. Although current therapeutic studies have majorly focused on the oncology field,^[85,86]^ the regulatory mechanisms of ncRNA are similarly considered as potential targets for male infertility treatment.^[87]^ For example, using anti-miRs and miRNA mimics to regulate miRNA expression levels,^[88]^ and developing specific lncRNA-targeting drugs,^[89]^ are novel strategies for male infertility.

miRNAs play a pivotal role in multiple stages of spermatogenesis, including the proliferation and differentiation of spermatogonial stem cells, as well as sperm cell maturation and division, directly impacting sperm quality and quantity.^[90]^ For instance, the production of reactive oxygen species (ROS), especially mitochondria-derived H_2_O_2_, fluctuates with dietary sugar levels, influencing miRNA expression.^[91]^

Notably, miRNA-221 is highly expressed in undifferentiated spermatogonial stem cells and is essential for maintaining their stemness; its impairment initiates cell differentiation and the loss of stemness.^[92]^ miRNA-221 also regulates spermatogenesis in mice by targeting molecules related to the SCF/c-Kit pathway.^[93]^ Other miRNAs, such as miR-449, are significantly upregulated during testicular development and early adult spermatogenesis, potentially influencing the E2F-pRb signaling pathway.^[94]^ Conversely, low miR-34c expression in certain patients may disrupt spermatogenesis.^[95]^ In NOA patients, miR-141 and miR-7-1-3p are also upregulated, potentially influencing spermatogenesis by downregulating Cbl and Tgfb2.^[96]^ Furthermore, miR-122, miR-141, and miR-200 exhibited significant changes in azoospermia and oligoasthenozoospermia patients,^[97]^ which makes them valuable as potential biomarkers.

Moreover, miRNAs linked to varicocele severity, sperm count, and surgical outcomes, such as miR-122, miR-181a, and miR-34c, have emerged as promising diagnostic and prognostic tools for varicocele.^[98]^ The expression levels of certain miRNAs, including miR-34c and miR-449b-5p, also correlate with the success rate of assisted reproductive technology, supporting their utility as predictive biomarkers for assisted reproductive technology outcomes.^[26]^

Due to their stability and ease of detection, miRNAs are highly suitable for rapid and noninvasive diagnosis of various diseases.^[99]^ Abnormal miRNA expression during spermatogenesis is linked to male infertility, underscoring their potential as biomarkers for evaluating spermatogenesis and diagnosing infertility.

While miRNAs have garnered significant research attention in diagnosis and treatment, other ncRNAs, including lncRNAs, circRNAs and piRNA, are also being investigated for their roles in disease diagnosis and treatment. For example, hypoxia-related lncRNAs (MIR210HG and MLLT4-AS1) were significantly elevated in sperm from infertile men with varicocele, correlating with ROS levels and lower sperm counts and motility.^[100]^

A genome-wide association study of 159 samples (83 with normal sperm and 76 with fertility issues) identified 6 lncRNAs associated with male infertility through interactions with miRNAs. Bioinformatics analyses revealed hypoxia response elements in their promoters, suggesting a role in gene regulation. These findings suggest that abnormal lncRNA regulation contributes to NOA, underscoring their relevance in spermatogenesis through the ceRNA network.^[101]^

Additionally, 7 differentially expressed circRNAs have been validated as potential upstream targets, with circRNA-miRNA-mRNA networks constructed to predict their functions.^[102]^ As miRNA sponges, circRNAs inhibit miRNA activity and regulate protein binding and gene transcription, leading to cell cycle arrest and spermatogonial apoptosis, thus impacting spermatogenesis.^[103]^ The primary mechanisms of piRNAs include maintaining germ cell function, regulating mRNA stabilit, and silencing transposons. Aberrations in piRNAs have been linked to cell cycle arrest, impaired spermatogenesis, reduced sperm motility, and abnormal sperm morphology in NOA,^[104]^ underscoring their essential role in reproductive health.

Although ncRNAs have exhibited great potential in the diagnosis and treatment of male infertility, numerous challenges must be overcome to achieve these results, including the accurate identification of ncRNA candidates and their targets, the precise delivery of therapeutic agents, and the management of complex data. Establishing global standards and addressing challenges are key to the development of reliable and effective treatments. Future studies will further explore the role of ncRNAs in gene regulation, epigenetic modification, and cell signaling to develop more precise diagnostic and therapeutic approaches, thereby bringing in new hope for male patients with infertility.

#### 4.2.5. Plants in crop improvement and abiotic stress

Food security is being challenged by climate change, population growth, and various abiotic and biotic pressures. With advancements in second-generation sequencing technologies, we could deeply explore and identify non-protein-coding parts of plant genomes, especially ncRNA, which play a key role in responding to environmental stresses such as drought, salinity, low and high temperature, thereby markedly driving crop improvement and stress tolerance.^[105,106]^ For example, lncRNAs, miRNAs, and phasiRNAs can serve as environmental stress sensors and mitigate male sterility and yield loss in plants.^[107]^ MiR408a serves as a negative regulator of drought response in maize, thus responding to drought stress by regulating the accumulation of reactive ROS in roots.^[108]^ ROS overproduction under various abiotic stress conditions leads to oxidative stress.^[109]^ MiR398 enhances tolerance to oxidative stress in plants, which is critical for counteracting various abiotic stresses.^[110]^

Salt stress significantly affects the growth, yield, and quality of plants. MiRNAs are known to be play a key role in the response to salt stress by regulating target genes. For example, under salt stress, transgenic plants had a higher seed germination rate, fresh weight, and root length, which reflects the regulatory importance of miRNAs.^[111,112]^ Moreover, plants with lncRNA973 knockdown exhibited poor tolerance under salt stress.^[113]^ LncRNA354 improved multi-stress tolerance in *Arabidopsis* by regulating stress response-associated gene expression.^[114]^ On comparing miRNA expression in plants, researchers found that miRNA families exhibit different expression patterns under different stress conditions.

In studies on photoperiod-sensitive male sterility, a lncRNA known as long day-specific male fertility-associated RNA played a regulatory role in rice. An adequate amount of long day-specific male fertility-associated RNA transcripts is essential for normal pollen development under long-day conditions.^[25]^ Moreover, ncRNAs play a remarkable role in regulating plant responses to heat stress. For instance, the ncRNA encoded by P/TMS12-1 produces a 21-nucleotide small RNA, which induces photoperiod-sensitive and thermosensitive sterility in japonica and indica rice varieties,^[29]^ respectively. Low-temperature stress, a major abiotic stress that reduces rice yield, is associated with lncRNA upregulation, which enhances cold resistance in plants by regulating cold-responsive genes.^[115]^ lncRNA-AIR significantly enhances the tolerance of *Arabidopsis* to various stresses by regulating the expression of abiotic stress response-related genes.^[116]^

Despite the remarkable progress in ncRNA studies on plant response to abiotic stresses, relatively limited work has been done in animal infertility. The ncRNA machinery in plants offers indirect implications for understanding animal infertility. For example, because of the role of miRNAs in regulating testicular function and spermatogenesis, cells may respond to external stresses and thus increase reproductive capacity.^[117]^ Future ncRNA-based breeding strategies are anticipated to optimize plant growth and stress tolerance to maximize crop yield even under climate change conditions, which is crucial for augmenting global food security.

## 5. Conclusion

This study contributes significantly to ncRNA research in infertility by conducting a comprehensive bibliometric analysis of animal and plant male sterility-related ncRNA studies from 2005 to 2023. Our analysis encompasses an in-depth examination of article distribution across countries, institutions, research scholars, and journals, along with co-citation patterns, keyword clustering, timelines, and burst analyses. The findings reveal 5 key future research directions: gene regulation, new technology applications, biological models, animal diagnostics and treatments, and crop improvement under abiotic stress. The study enhances our understanding of the role of ncRNAs as biomarkers in animal studies, underscoring their significance in reproductive development, and highlights plant resilience to environmental stress. This work identifies essential areas for further exploration, promoting clinical applications in male infertility and supporting agricultural advancements.

## 6. Strengths and limitations

Despite its contributions, this study has certain limitations. It includes only English-language articles and reviews from the WoSCC database, which may introduce bias and limit the scope of findings. Additionally, for research convenience, this study divided the research subjects into animals and plants, combining human and animal studies. This approach may have impacted the accuracy of conclusions, and future studies should separately address animals, plants, and humans for precise insights. Nonetheless, the study’s conclusions remain scientifically valid and informative. As sequencing technologies and bioinformatics advance, further understanding of ncRNA functions will likely drive the development of ncRNA-based biomarkers and therapeutic strategies. This research not only provides valuable insights for clinical practice in male infertility but also contributes to agricultural innovation, supporting efforts to address global health and food security challenges.

## Acknowledgments

The authors would like to thank National Natural Science Foundation of China, China Association of Chinese Medicine, National Advantageous Specialty Construction Project of Traditional Chinese Medicine. The authors would like to thank all the reviewers who participated in the review, as well as MJEditor (www.mjeditor.com) for providing English editing services during the preparation of this manuscript.

## Author contributions

**Conceptualization:** Xiangyu Wang, Shuxi Zhou, Haobin Zhao, Xiao Li.

**Formal analysis:** Xiangyu Wang, Shuxi Zhou, Haobin Zhao.

**Funding acquisition:** Zu long Wang, Xiao Li.

**Investigation:** Mingzhao Zhang, Baojun Ju, Xiangyu Wang, Shuxi Zhou, Haobin Zhao.

**Methodology:** Mingzhao Zhang, Baojun Ju, Zu long Wang.

**Resources:** Xiao Li.

**Software:** Mingzhao Zhang.

**Supervision:** Xiao Li.

**Validation:** Mingzhao Zhang, Baojun Ju.

**Writing – original draft:** Mingzhao Zhang.

**Writing – review & editing:** Mingzhao Zhang, Zu long Wang, Xiao Li.
